# Kidney and Bladder Transplantation: Advances, Barriers, and Emerging Solutions

**DOI:** 10.3390/medicina61061045

**Published:** 2025-06-05

**Authors:** Gani Kuttymuratov, Timur Saliev, Ardak Ainakulov, Askar Ayaganov, Kuat Oshakbayev, Daulet Zharassov, Abdurakhman Tuleuzhan, Nurlybek Uderbayev

**Affiliations:** 1 Department of Multidisciplinary Surgery, University Medical Center, Nazarbayev University, Astana 010000, Kazakhstan; gani.kt@umc.org.kz; 2 Institute for Fundamental and Applied Medical Research, S.D. Asfendiyarov Kazakh National Medical University, Almaty 050000, Kazakhstan; 3 Department Urology and Kidney Transplantation, University Medical Center, Nazarbayev University, Astana 010000, Kazakhstan; 4 Department of Anesthesiology, Resuscitation and Intensive Care, University Medical Center, Nazarbayev University, Astana 010000, Kazakhstan; 5 Internal Medicine Department, University Medical Center, Nazarbayev University, Astana 010000, Kazakhstan; 6 Department of General Surgery, West Kazakhstan Medical University named after Ospanov Marat, Aktobe 030012, Kazakhstan

**Keywords:** urogenital transplantation, kidney graft, bladder transplantation, tissue engineering, regenerative medicine, xenotransplantation, immunosuppression, organ replacement

## Abstract

Urogenital transplantation has emerged as a ground-breaking field with the potential to revolutionize the treatment of end-stage organ failure and congenital or acquired defects of the kidney and urinary bladder. This review provides a comprehensive analysis of the current state, clinical experiences, and experimental progress in kidney and bladder transplantation, with a particular focus on immunological, surgical, and ethical challenges. While kidney transplantation is now a well-established procedure offering improved survival and quality of life for patients with chronic renal failure, bladder transplantation remains in the experimental phase, facing hurdles in vascularization, tissue integration, and functional restoration. Recent advancements in tissue engineering, regenerative medicine, and immunosuppressive strategies are critically discussed, highlighting their role in shaping the future of urogenital grafts. This review also explores xenotransplantation and bio-artificial organ development as promising frontiers. Continued interdisciplinary research is essential to overcome the current limitations and enable routine clinical application of bladder transplantation while optimizing outcomes in kidney grafts.

## 1. Introduction

Kidney and bladder transplantation have become transformative interventions for restoring function in patients with severe renal or bladder failure, offering a vital solution for organ replacement in cases of end-stage dysfunction [1,2]. As a critical medical advancement, this field aims to improve both survival and quality of life in individuals suffering from organ failure due to congenital anomalies, trauma, or disease [3]. Among urogenital transplants, kidney transplantation is the most widely practiced and clinically successful, offering patients with end-stage renal disease (ESRD) a viable alternative to dialysis [1,4]. This procedure has significantly enhanced millions of patients’ life expectancies and overall well-being worldwide. Despite its success, kidney transplantation faces challenges such as donor organ shortage, limited graft longevity, and lifelong immunosuppression with associated infection and cancer risks [5,6].

In contrast, bladder transplantation remains largely experimental and has yet to achieve clinical application [7]. Bladder transplantation is considered for patients with dysfunction from congenital anomalies, neurogenic bladder, or bladder loss due to cancer or trauma. Existing treatments like augmentation and diversion often fail to restore normal function, significantly impacting quality of life [8]. While a successful bladder transplant could offer an ideal solution by restoring normal urinary storage and voiding function, several formidable challenges stand in the way of its clinical implementation [3,9]. Unlike kidney transplantation, bladder transplantation requires complex vascular and neural integration to restore functional innervation and coordinated muscle activity [8,10]. Additionally, the immune response to a transplanted bladder remains poorly understood, further complicating efforts to develop viable immunosuppressive strategies.

Despite challenges, urological transplantation advances through improved surgery, tissue engineering, and immunology, with xenotransplantation offering a potential solution to organ shortages [8,11]. Bioengineered organs from autologous cells offer personalized grafts with lower rejection risk [12,13]. Furthermore, the development of novel immunosuppressive regimens, including tolerance-inducing protocols and cell-based therapies, may mitigate the need for lifelong immunosuppression and reduce the associated complications [14,15].

Despite these exciting prospects, the translation of these innovations from experimental models to clinical practice remains fraught with scientific, ethical, and logistical challenges. Ethical concerns surrounding xenotransplantation, including cross-species disease transmission and public acceptance, must be addressed before widespread implementation can be considered [16,17]. Similarly, bioengineered organs require further refinement to achieve structural and functional equivalency to native tissues. Moreover, financial and regulatory barriers continue to shape the feasibility of these cutting-edge approaches, influencing the pace at which they progress toward clinical application [17,18].

This review mainly focuses on kidney and bladder transplantation, highlighting recent advances and ongoing challenges in kidney transplantation while exploring the emerging field of bladder grafting. By analyzing current research, clinical trials, and technological developments, it outlines potential future directions. Understanding both the progress achieved and the barriers that remain is essential for evaluating the role of urological transplantation in advancing reconstructive urology and transplant medicine.

## 2. Kidney Transplantation: Achievements and Ongoing Challenges

Kidney transplantation is the most common and successful solid organ transplantation worldwide, offering significant improvements in both survival and quality of life for patients suffering from end-stage renal disease (ESRD) [1]. Over the past several decades, the field has undergone remarkable advancements, driven by innovations in surgical techniques, immunosuppressive therapies, organ preservation methods, and donor–recipient matching strategies [19,20]. These improvements have enhanced short- and long-term transplant outcomes, yet several persistent challenges continue to limit the widespread success and accessibility of kidney transplantation [21,22].

One of the most critical advancements in the field has been the refinement of surgical techniques, leading to improved graft survival rates and reduced postoperative complications [23,24,25]. The standard approach involves the implantation of the donor kidney in the recipient’s iliac fossa, optimizing vascular anastomosis and facilitating monitoring [1]. Minimally invasive and robotic-assisted surgical techniques are also being increasingly adopted, particularly in living donor nephrectomy, reducing morbidity and encouraging more individuals to participate in organ donation programs. Alongside surgical advancements, post-transplant care has significantly improved, incorporating enhanced monitoring protocols that utilize non-invasive biomarkers, genetic profiling, and advanced imaging techniques [26]. These innovations, coupled with the integration of artificial intelligence in predictive analytics, allow for the early detection of rejection and graft dysfunction, enabling clinicians to refine immunosuppressive regimens and personalize treatment strategies [27,28].

Table 1 summarizes the recent advancements, ongoing challenges, and potential solutions in the field of kidney transplantation. It highlights key areas of progress, including improvements in surgical techniques, immunosuppressive therapies, and organ preservation methods. At the same time, it addresses persistent issues such as organ shortage, graft rejection, and long-term graft survival. Furthermore, the table outlines the innovative strategies being explored to overcome these challenges, including regenerative medicine, precision immunotherapy, and advancements in xenotransplantation and bio-artificial kidneys (Table 1).

The success of kidney transplantation largely depends on immunosuppressive therapy, which prevents the recipient’s immune system from attacking the transplanted organ [29,30]. The introduction of calcineurin inhibitors, mTOR inhibitors, and biologic agents such as monoclonal antibodies has significantly reduced acute rejection episodes. However, the long-term use of immunosuppressive drugs remains a double-edged sword, as while they prevent graft loss, they also predispose patients to opportunistic infections, malignancies, and metabolic disorders such as diabetes and hypertension [31,32]. This has led to the exploration of personalized immunosuppressive strategies that aim to minimize adverse effects while maintaining graft viability [33]. Precision medicine, particularly pharmacogenomics, is now enabling more tailored immunosuppressive regimens based on individual genetic predisposition, optimizing both safety and efficacy.

**Table 1:** Summary of recent advancements, ongoing challenges, and potential solutions in the field of kidney transplantation.

Domain	Key Achievements	Current Challenges	Potential Solutions	Ref.
Surgical Techniques	Standardized implantation in iliac fossa	Limited global access to advanced surgical tools and training	Expand surgical education programs	[34]
	Adoption of minimally invasive and robotic-assisted procedures		Increase investment in robotic systems in transplant centers	[35]
Post-Transplant Monitoring	Use of biomarkers, imaging, and AI analytics	Lack of standardized, personalized monitoring protocols	Develop evidence-based monitoring algorithms	[36]
	Early detection of graft dysfunction		Integrate AI tools in routine follow-up care	[37]
Immuno- suppressive Therapy	Reduced acute rejection via calcineurin inhibitors, mTOR inhibitors, and biologics	Long-term toxicity: infections, malignancies, metabolic complications	Advance immune tolerance strategies	[38]
	Rise of pharmacogenomic tailoring	Lifelong dependency	Invest in next-generation, low-toxicity immuno- suppressants	[39]
Organ Preservation	Hypothermic and normothermic perfusion improves graft viability and function	High costs and limited availability in resource-poor settings	Scale up cost-effective machine perfusion systems	[40]
			Enhance perfusion fluids with therapeutic agents (e.g., anti-inflammatory drugs)	[41]
Donor–Recipient Matching	Precision genetic and immune profiling	Persistent chronic allograft dysfunction and late rejection	Improve early detection of subclinical rejection	[42]
	Virtual cross-matching		Research into individualized immunologic risk profiling	[43]
Immune Tolerance Strategies	Experimental success in chimerism and regulatory T-cell therapy	Lack of clinical validation and scalability	Support clinical trials and translational research	[44]
			Combine cellular therapies with targeted immunomodulation	[45]
Alternative Organ Sources	Gene-edited porcine kidneys showing promise	Immunologic risks, zoonoses, technical complexity, and ethical debates	Strengthen ethical/regulatory frameworks	[46]
	Advances in 3D bioprinting and organ scaffolding		Promote R&D in scaffold engineering, vascularization, and stem cell biology	[47]
Ethical and Societal Considerations	Growing consensus on ethical organ allocation	Inequities in access and public hesitation toward emerging technologies	Implement equitable allocation policies	[48]
			Increase public education and donor awareness	[49]
Economic and Logistical Factors	Efficient transplant systems in high-income countries	High costs of immunosuppression, organ procurement, and post-op care in low-resource areas	Global partnerships and funding mechanisms	[50]
			Encourage local production of generics and simplified care protocols	[51]

A crucial factor influencing transplantation outcomes is the preservation of donor organs before implantation [52]. Traditional static cold storage, while widely used, has limitations in reducing ischemia–reperfusion injury. Consequently, newer techniques such as hypothermic and normothermic machine perfusion have demonstrated superiority in preserving graft quality [53,54]. Hypothermic perfusion actively circulates a cold perfusate through the donor kidney, reducing ischemic damage, particularly in kidneys from extended-criteria donors and donation-after-circulatory-death donors. Normothermic perfusion, on the other hand, maintains the kidney under near-physiological conditions, delivering oxygen and nutrients and allowing pre-transplant viability assessments [40]. These methods not only enhance immediate graft function but also reduce delayed graft function, a common complication associated with ischemic injury. Future advancements in organ preservation may incorporate therapeutic agents into perfusion solutions, such as anti-inflammatory drugs or gene therapy vectors, to further enhance graft longevity.

Beyond preservation, significant strides have been made in donor–recipient matching, an essential factor in ensuring successful transplantation. Advances in genetic and immune profiling now allow for more precise compatibility predictions, reducing the likelihood of rejection [55,56]. Virtual cross-matching, using computational models, enables early assessments of immune compatibility, allowing a better allocation of available organs. However, despite these improvements, chronic allograft dysfunction remains a major concern, with rejection, interstitial fibrosis, and tubular atrophy being leading causes of late-stage graft failure [57,58]. Researchers are actively investigating immune tolerance induction strategies to overcome these challenges, including chimerism-based approaches, where donor bone marrow or hematopoietic stem cells are co-transplanted with the kidney to promote immune adaptation [59,60]. Additionally, regulatory T-cell therapy and costimulation blockade therapies are being explored as potential methods to modulate immune responses and prevent chronic rejection without the need for lifelong immunosuppression [61,62].

One of the most pressing issues in kidney transplantation is the persistent shortage of donor organs, with demand far exceeding supply. This disparity has led to an increased exploration of alternative sources, particularly xenotransplantation and bioengineered kidneys [63]. Recent advancements in xenotransplantation research have significantly accelerated the prospects of porcine kidney grafts for human clinical application. Several pivotal studies have demonstrated not only the technical feasibility but also the physiological functionality of genetically engineered pig kidneys in human models, laying the groundwork for future clinical trials [64,65].

Porrett et al. (2022) conducted the first clinical-grade porcine kidney xenotransplant in a human decedent model, representing a milestone in translational research [66]. Their study successfully demonstrated the technical feasibility of porcine kidney transplantation into humans, achieving hemodynamic stability and maintaining vascular integrity without hyperacute rejection over 74 h. While the grafts produced variable urine output, creatinine clearance did not recover, likely influenced by brain death-related factors and microvascular injury. Importantly, no porcine retrovirus transmission or chimerism was observed, marking a significant safety milestone.

Complementing these findings, Montgomery et al. (2022) reported on two cases of pig-to-human kidney xenotransplantation using genetically modified pigs with alpha-1,3-galactosyltransferase gene knockout and autologous thymic tissue [67]. Their results were encouraging: both xenografts began producing urine immediately post-reperfusion, with the eGFR and creatinine levels improving notably over 54 h. Biopsies revealed no signs of hyperacute or antibody-mediated rejection, and the urine output exceeded that of the native kidneys, indicating robust initial graft function.

Expanding on the physiological impact, Judd et al. (2024) investigated the capacity of gene-edited pig kidneys to maintain human physiological homeostasis in a brain-dead decedent over seven days [68]. The study measured critical parameters such as the renin-angiotensin–aldosterone system (RAAS), parathyroid hormone levels, and markers of water and electrolyte balance. The xenografts demonstrated sustained urine production, functional aquaporin 2 channel expression, and a creatinine clearance ranging from 90–240 mL/min. These results provided strong evidence of the xenografts’ ability to support essential renal endocrine and excretory functions in a human physiological environment.

Further insights into the endocrine functionality of porcine kidney xenografts were provided by Firl et al. (2023) in a study involving 17 cynomolgus macaques [69]. Their analysis showed that while xenografts maintained moderate growth and demonstrated a limited RAAS pathway contribution, they induced parathyroid hormone-independent hypercalcemia and hypophosphatemia. These findings highlight potential metabolic complications that require careful monitoring in future human trials.

Rodger and Cooper (2022) provided a comprehensive review of xenotransplantation progress, underscoring the scientific, ethical, and public health considerations surrounding clinical application [70]. They emphasized that despite technological breakthroughs, challenges such as long-term graft survival, immunological compatibility, and public acceptance remain significant hurdles. Collectively, these studies underscore the transformative potential of porcine kidney xenotransplantation. The convergence of genetic engineering, immunomodulation, and advanced surgical techniques has enabled remarkable progress in overcoming hyperacute rejection and achieving functional graft performance. However, persistent challenges related to metabolic regulation, long-term graft viability, and immunological integration necessitate continued interdisciplinary research and cautious progression to clinical trials.

The use of CRISPR-Cas9 gene editing has enabled scientists to modify porcine organs to reduce immunogenicity and prevent hyperacute rejection [71]. However, concerns regarding zoonotic infections, long-term immune responses, and ethical considerations surrounding the use of animal organs remain significant obstacles [46,72]. Meanwhile, bioengineered and 3D-printed kidneys represent another exciting frontier in transplantation research. Scientists are developing artificial kidneys by using decellularized scaffolds repopulated with human stem cells, aiming to recreate functional nephron structures. Advances in 3D bioprinting have also allowed for the fabrication of nephron-like structures, although achieving full organ functionality remains a challenge [73]. The integration of vascular networks and ensuring the long-term viability of engineered tissues are critical hurdles that need to be addressed before bioengineered kidneys become a viable clinical reality.

Despite the scientific and technological advancements in kidney transplantation, ethical, financial, and logistical challenges persist. Ethical considerations include equitable organ allocation, the risks and benefits of xenotransplantation, and the societal implications of emerging biotechnologies [72]. Financially, the high costs of immunosuppressive drugs, organ procurement, and post-transplant care create accessibility issues, particularly in lower-income regions. Additionally, disparities in access to transplantation remain a concern, with socioeconomic factors influencing patient eligibility and organ availability [74,75]. Addressing these challenges requires interdisciplinary collaboration between transplant surgeons, immunologists, bioengineers, policymakers, and ethicists to develop sustainable, ethically sound, and widely accessible transplantation strategies.

Kidney transplantation has made extraordinary strides, becoming the gold standard for ESRD treatment and significantly improving patient outcomes. Advances in surgical techniques, immunosuppressive therapies, organ preservation, and donor–recipient matching have collectively contributed to better graft survival rates and long-term success [23,76].

However, persistent challenges, ranging from chronic allograft dysfunction to organ shortages and ethical dilemmas, continue to limit the full potential of transplantation. Ethical concerns remain particularly significant in areas such as living donor programs, commercialization risks, and equitable access to care. Martinelli et al. (2024) offer a comprehensive overview of the psychosocial and ethical issues surrounding living donor kidney transplantation (LDKT), highlighting the psychological, interpersonal, and social impacts on both donors and recipients [46]. Their review underscores the ethical complexities of expanding living donation practices, including paired exchange programs, Samaritan donations, and pre-emptive transplants, all analyzed through the lens of biomedical ethics principles: autonomy, beneficence, non-maleficence, and justice.

The commercialization of kidney transplantation through living-unrelated paid donation (LURpD) remains a controversial issue. Sever et al. (2022) critically discuss the medical, ethical, and social dilemmas posed by paid donor programs, warning of the dangers of organ commodification, black market exploitation, and the erosion of public trust [77]. Despite widespread disapproval, they note that up to 10% of global transplants may involve illicit practices. The authors advocate for strict regulations and ethical oversight, urging the nephrology community to prioritize accessible and equitable kidney care to counteract the drivers of organ trafficking and transplant tourism.

Another dimension of ethical concern relates to vulnerable populations, such as migrants, who often face barriers to accessing kidney replacement therapies. Martin et al. (2022) explore the ethical complexities in providing transplantation to migrant populations, particularly in contexts where residency status and citizenship influence eligibility for treatment [78]. Their analysis underscores the tension between national self-sufficiency goals and the moral obligation to provide equitable care while also addressing the heightened vulnerability of migrants to organ trafficking and exploitation.

Xenotransplantation has also entered ethical debates as a potential solution to organ shortages. Bayliss (2022) discusses the practical and ethical considerations of transplanting pig kidneys into humans [79]. While recent proof-of-concept surgeries have demonstrated feasibility, unresolved concerns regarding immunologic safety, infectious risks, long-term outcomes, and cost-effectiveness remain. The author emphasizes the need for carefully designed human trials and robust ethical frameworks to ensure the responsible implementation of xenotransplantation in clinical practice.

Collectively, these studies highlight that beyond clinical success, kidney transplantation raises profound ethical questions that intersect with societal values, healthcare equity, and global regulatory challenges. Addressing these dilemmas requires continuous ethical reflection, policy development, and international collaboration to safeguard both donor and recipient rights while ensuring access to life-saving therapies.

## 3. Bladder Transplantation: Emerging Prospects

Bladder transplantation, unlike kidney transplantation, remains an experimental endeavor with numerous scientific and clinical challenges [22]. The primary obstacles stem from the anatomical and functional complexity of the bladder, which involves intricate vascular supply and dense neural networks that regulate both urinary storage and voiding functions [80]. Unlike other solid organs, the bladder must not only be adequately perfused but also maintain its compliance, elasticity, and sensory–motor control to ensure normal urinary function [1]. The success of a bladder transplant depends on the precise restoration of blood supply, functional nerve integration, and the prevention of complications such as graft fibrosis, impaired detrusor muscle function, and immune-mediated rejection.

One of the greatest hurdles in bladder transplantation is the need for full neural re-innervation. The bladder’s dual innervation autonomic (sympathetic and parasympathetic) and somatic (pudendal nerve control) governs both involuntary and voluntary urinary control [9]. Even if vascular anastomosis is achieved, inadequate nerve regeneration may lead to detrusor underactivity, urinary retention, or incontinence. While peripheral nerve grafting techniques and neurotrophic factor therapy have been explored to promote neural regeneration, achieving complete functional recovery remains a formidable challenge [80]. The complexity of neural pathways means that even if transplanted bladder tissue remains structurally intact, functional integration with the recipient’s nervous system may be incomplete, limiting its practical application [81].

**Figure 1:**
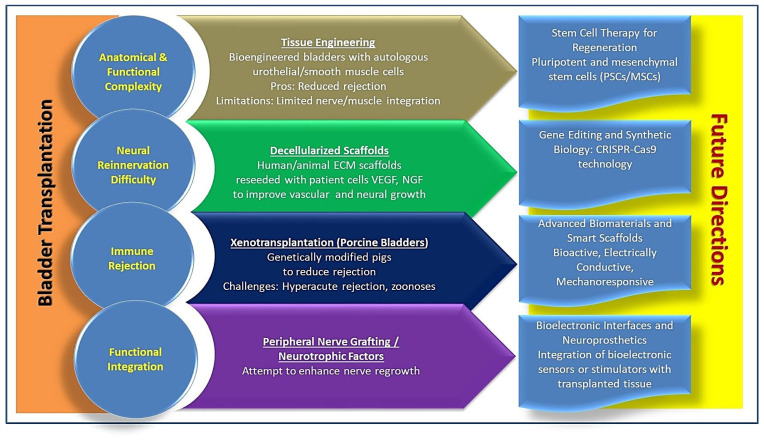
The figure summarizes key challenges in bladder transplantation, including anatomical and functional complexity, neural re-innervation difficulty, immune rejection, and functional integration. Potential solutions include tissue engineering with autologous cells to reduce rejection, decellularized scaffolds reseeded with patient-derived cells to enhance vascular and neural growth, xenotransplantation using genetically modified porcine bladders to minimize rejection risks, and peripheral nerve grafting to promote nerve regrowth. Future directions highlight emerging technologies such as stem cell therapy, gene editing (CRISPR-Cas9), advanced bioactive scaffolds, and bioelectronics interfaces to improve graft function and integration. These strategies aim to address current barriers and advance bladder transplantation towards clinical application.

Despite these obstacles, advances in regenerative medicine and tissue engineering have provided new hope for bladder reconstruction [82] (Figure 1). Recent developments have focused on the creation of bioengineered bladder constructs, utilizing autologous cells seeded onto biodegradable scaffolds [1]. In preclinical and early experimental studies, tissue-engineered bladders composed of urothelial and smooth muscle cells derived from the patient’s own tissue have shown promise in restoring partial urinary function. By using the patient’s own cells, the risk of immune rejection is significantly reduced, eliminating the need for lifelong immunosuppression. However, while these engineered bladders have demonstrated structural feasibility, long-term functionality, particularly in terms of muscle contractility and nerve integration, remains a major concern.

Another promising avenue in bladder transplantation research involves the use of decellularized bladder matrices from human or animal sources as scaffolds for cell reseeding (Figure 1). This approach retains the native extracellular matrix (ECM) architecture, providing a natural template for vascular and neural regeneration [83,84]. Decellularized bladders have been investigated in preclinical settings, showing improved biocompatibility and reduced immune responses [85,86]. When reseeded with autologous urothelial and smooth muscle cells, these scaffolds have demonstrated the potential to develop functional bladder tissue. Furthermore, incorporating growth factors such as vascular endothelial growth factor (VEGF) and nerve growth factor (NGF) has been explored to enhance angiogenesis and neural integration, facilitating more effective graft adaptation (Figure 1) [87,88]. However, while animal models have shown some success, translating these findings into human clinical applications remains challenging due to variability in host immune response, fibrosis risk, and limited long-term data on functional restoration.

The potential of xenotransplantation as a solution to bladder graft shortages has also been investigated, particularly through the use of porcine bladders (Figure 1) [89]. Pig organs have been widely considered for transplantation due to their anatomical similarity to human tissues, and genetically modified pigs have been developed to reduce immune rejection [90]. However, progress in bladder xenotransplantation has been considerably slower compared to kidney xenotransplantation [91,92]. One of the main challenges is the high risk of hyperacute rejection due to preformed antibodies targeting porcine antigens, as well as concerns regarding zoonotic disease transmission (Figure 1) [89,93]. While genetic engineering strategies such as the knockout of α-Gal epitopes and the introduction of human complement regulatory proteins have helped mitigate some of these immunological barriers, further research is needed to ensure long-term graft survival and functionality.

Beyond the biological and immunological challenges, the ethical and practical considerations of bladder transplantation must also be addressed [72,74]. Unlike kidney transplants, which offer a clear survival benefit for patients with end-stage renal disease, bladder transplantation is primarily aimed at improving quality of life. Many patients with bladder dysfunction currently rely on urinary diversion techniques, such as ileal conduit diversion or neo-bladder reconstruction using bowel segments. While these methods are associated with complications such as infection, metabolic disturbances, and impaired continence, they remain the standard of care in the absence of viable bladder transplantation options. Therefore, the potential benefits of bladder transplantation must be carefully weighed against the risks associated with surgery, immunosuppression, and uncertain long-term outcomes.

Recent advances in experimental models and surgical techniques have significantly expanded the possibilities for urinary bladder transplantation, addressing a long-standing clinical need for alternatives to gastrointestinal neo-bladder reconstruction. Wang et al. introduced a novel orthotopic mouse bladder transplantation model in which the donor bladder, along with its vascular pedicle, is transplanted into the recipient after partial cystectomy [94]. This model demonstrated more than an 80% six-month survival, and, importantly, the recipient mice exhibited normal bladder function and structure comparable to controls, as confirmed by cystometric analysis and histology. The model also allowed for the study of immune rejection, as allogeneic transplants showed early acute rejection, highlighting its utility for both functional and immunological research.

Complementing these findings, Jundziłł et al. developed a heterotopic vascularized bladder transplantation model in rats, performing the transplant in the right groin vessels [95]. Their study achieved an 80% graft survival rate at one week, with no significant loss of bladder tissue or evidence of inflammation in surviving grafts. This technique offers a new approach to bladder transplantation research, though its heterotopic nature may limit direct functional assessment compared to orthotopic models.

In the context of translational and clinical application, Nassiri et al. reported on preclinical studies of robotic bladder autotransplantation using vascularized composite allografts in porcine models, human cadavers, and brain-dead donors [96]. Their work demonstrated the technical feasibility of robotic retrieval, back-table preparation, and transplantation, achieving successful reperfusion and sustained bladder vascularity. The use of indocyanine green immunofluorescence confirmed graft viability, and the operative times improved with experience. These results represent a critical step toward first-in-human clinical trials, although the complexity of deep pelvic vascular anatomy and the need for advanced surgical expertise remain significant challenges.

Further supporting the feasibility of bladder transplantation, Gargollo et al. conducted cadaveric studies to map the vascular anatomy and perform mock transplants using external iliac vessels for vascular anastomosis [97]. Their findings confirmed that bladder vascularized composite allograft transplantation is technically and anatomically possible, laying the groundwork for ongoing phase 1 clinical trials. However, these studies did not address the immunologic or long-term functional outcomes, which are essential for clinical success.

A broader review by Shen et al. emphasized the diversity of animal models available for studying bladder function and dysfunction, ranging from rodents to large mammals [98]. They highlighted that while small animal models are valuable for mechanistic and immunologic studies, they may not fully replicate the complexity of human bladder physiology and pathology. Larger animal and robotic models, as demonstrated by Nassiri et al., are necessary to bridge this translational gap and address the technical and functional challenges of clinical bladder transplantation.

In summary, orthotopic and heterotopic animal models have established the functional viability and immunologic challenges of bladder grafts, while preclinical and cadaveric studies have advanced the technical feasibility of transplantation in humans. The next steps will require standardization of surgical techniques, integration of robust immunosuppressive protocols, and comprehensive functional assessments in large-animal models to ensure safe and effective clinical translation.

A successful bladder transplant requires a multidisciplinary team, including urologists, transplant surgeons, bioengineers, neuroscientists, and immunologists. Key challenges remain in improving vascularization, neural integration, and immune tolerance. Regenerative medicine and bioengineering, including stem cell therapy, gene editing, and advanced biomaterials, offer promising solutions. Before clinical use, extensive preclinical studies and well-designed trials are essential. Though still in its early stages, ongoing research suggests that bladder transplantation could become a viable treatment in the coming decades, transforming care for severe bladder dysfunction.

## 4. Immunological Challenges and Graft Survival

Transplantation of the kidney and bladder presents unique and formidable immunological challenges, particularly in preventing both acute and chronic graft rejection [99]. While kidney transplantation has benefited from decades of research and the development of increasingly sophisticated immunosuppressive protocols, bladder transplantation remains in its infancy, with many unresolved immunological complexities [100]. The bladder’s distinct physiological environment, characterized by continuous exposure to urine, host microbiota, and dynamic urothelial turnover, poses additional hurdles in maintaining graft viability while preventing immune-mediated damage.

Acute rejection in organ transplantation is primarily mediated by T-cell activation, which triggers an inflammatory cascade that leads to graft injury and potential failure (Table 2) [100]. This process is driven by antigen-presenting cells recognizing alloantigens and initiating cytotoxic responses [59]. Current immunosuppressive strategies, including calcineurin inhibitors, corticosteroids, mTOR inhibitors, and biologic agents targeting costimulatory pathways (such as CTLA-4 inhibitors), have been effective in reducing the acute rejection rates in kidney transplants [101,102]. However, these therapies often carry significant side effects, including nephrotoxicity, metabolic disturbances, and an increased risk of opportunistic infections [103,104]. The challenge in bladder transplantation is to balance effective immunosuppression while preserving urothelial integrity, reducing systemic toxicity, and maintaining the bladder’s functional properties.

Chronic rejection remains a major barrier to long-term graft survival, particularly due to immune-mediated fibrosis and progressive vascular injury. In the kidney, chronic allograft dysfunction is marked by interstitial fibrosis and tubular atrophy, while in the bladder, prolonged immune responses can lead to fibrosis, detrusor muscle dysfunction, and compromised urinary storage and voiding function [105,106]. Chronic rejection often involves a complex interplay of T-cell and B-cell responses, complement activation, and alloantibody-mediated injury (Table 2). Innovations such as costimulation blockade (e.g., belatacept) and regulatory T-cell (Treg) therapies have shown promise in promoting immune tolerance and reducing the long-term impact of alloimmune responses [107,108]. However, their efficacy in bladder transplantation remains largely untested in clinical settings.

A significant area of innovation in transplantation immunology is the development of localized immunosuppressive strategies that minimize systemic exposure and associated toxicities. In the context of bladder transplantation, localized drug delivery systems such as intravesical immunomodulatory agents offer an attractive approach to controlling immune activation directly at the graft site [109,110]. Studies have explored the use of nanoparticle-based delivery systems and hydrogels for a sustained intravesical release of immunosuppressants, aiming to achieve localized immune regulation while avoiding the systemic side effects of traditional regimens [111,112]. Additionally, the potential for gene-editing technologies, such as CRISPR-based interventions, to modify donor bladder cells for reduced immunogenicity presents an exciting avenue for future research (Table 2).

Advances in biomarker development are also playing a crucial role in early rejection detection and personalized transplant management. Non-invasive biomarkers, such as urine-based cytokines, donor-derived cell-free DNA (dd-cfDNA), and proteomics, are being investigated for their ability to predict rejection episodes before clinical symptoms manifest (Table 2) [113,114]. In kidney transplantation, dd-cfDNA has already been validated as a promising tool for monitoring graft health, and similar approaches are being explored for bladder transplants [115,116]. The integration of machine learning algorithms to analyze complex biomarker data could further enhance early detection and intervention strategies, ultimately improving long-term graft survival rates.

Beyond drugs and diagnostics, regenerative medicine and tissue engineering offer new ways to address the immunological challenges in bladder transplantation. Using autologous stem cells with biocompatible scaffolds and bioengineered bladder constructs may lower the need for donor organs and reduce immune rejection.

Decellularized bladder matrices, reseeded with a patient’s cells, could create functional grafts with minimal immune response, decreasing the need for lifelong immunosuppression. Future transplantation should focus on personalized immunosuppressive regimens. By using genetic, epigenetic, and biomarker data, precision medicine can help tailor drug choices and dosing, improving outcomes and reducing side effects. Addressing broader issues like healthcare access, transplant care availability, and patient education is also vital to ensure fair and successful treatment for all.

**Table 2:** Key aspects of immunological challenges and graft survival in urological transplantation, including clinical implications, current challenges, and possible solutions.

Aspect	Description	Clinical Implications	Challenges	Solutions/ Innovations	Ref.
Physiological Environment	Bladder exposed to urine, microbiota, and high urothelial turnover	Increases risk of infection, immune activation, and graft failure	Difficult to maintain immune balance while preserving functional urothelium	Tissue-compatible biomaterials; localized immunosuppression	[3]
Acute Rejection	T-cell-mediated inflammation from recognition of alloantigens	Graft damage, inflammation, and possible early transplant loss	Overactivation of the immune response; systemic toxicity from drugs	Calcineurin inhibitors, corticosteroids, CTLA-4 blockers; localized drug delivery	[7]
Chronic Rejection	Fibrosis, vascular injury, detrusor dysfunction from prolonged immune response	Long-term graft failure, loss of bladder compliance, incontinence	Hard to detect early; limited reversibility	Costimulation blockers (e.g., belatacept), Treg therapy	[117]
Systemic Immuno suppression	Drugs used in kidney transplants are also applied here, but with limitations	Risk of nephrotoxicity, metabolic issues, infections	Side effects undermine long-term safety	Development of bladder-specific regimens; localized delivery platforms	[118]
Localized Immuno suppression	Intravesical delivery via nanoparticles, hydrogels	Targets the bladder directly, reduces systemic exposure	Drug penetration and duration: clinical translation	Sustained-release vehicles; targeted immunosuppressants	[119]
Gene Editing	CRISPR is used to reduce the immunogenicity of donor cells	Potential for creating hypoimmunogenic bladder tissue	Ethical, safety, and technical issues in editing human grafts	CRISPR/Cas9-modified donor tissues; ongoing safety trials	[120]
Biomarkers	Urine cytokines, dd-cfDNA, and proteomics are used for early rejection detection	Enables non-invasive, proactive transplant monitoring	Validation for bladder application; complex interpretation	Integrate with machine learning for predictive modelling	[121]
Machine Learning	Analyzes complex biomarker datasets	Enhances early detection, enables personalized immunosuppression	Requires large, high-quality datasets	AI-powered decision tools for transplant monitoring	[122]
Regenerative Medicine	Use of stem cells and biocompatible scaffolds to engineer low-immunogenic grafts	Reduces immune response; improves integration	Ensuring muscle function and innervation	Autologous cell seeding, scaffold optimization, and hybrid tissue constructs	[7]
Xeno transplantation	Use of porcine bladder tissue modified to reduce immune recognition	May address graft shortages	Hyperacute rejection; zoonotic risk	Knockout of α-Gal; human complement regulators; pathogen screening	[8]
Precision Medicine	Tailored regimens based on genetic/biomarker profiles	Safer, more effective immunosuppression for each patient	Complex integration into clinical workflow	Use of pharmacogenomics, personalized dosing algorithms	[123]
Health System Factors	Address disparities in access to advanced transplant care	Equity in outcomes and long-term support	Unequal access, lack of education	Policy interventions, public education, and funding access initiatives	[124]

## 5. Simultaneous Bladder–Kidney Transplantation

The combination of bladder dysfunction and end-stage renal disease (ESRD) presents a significant clinical challenge, necessitating a multidisciplinary approach to optimize transplant outcomes. While kidney transplantation remains the gold standard treatment for ESRD, its long-term success is closely linked to the functional status of the lower urinary tract, particularly the bladder. In patients with severe bladder dysfunction, including those with neurogenic bladder, reduced bladder compliance, or severely reduced capacity, the dysfunctional bladder can pose a serious threat to kidney graft survival. Consequently, simultaneous bladder–kidney transplantation (SBKT) has emerged as a potential therapeutic strategy in selected patients.

The need for simultaneous bladder and kidney transplantation arises from the fact that a dysfunctional bladder can severely compromise the success of renal transplantation. High-pressure, non-compliant bladders can lead to upper urinary tract deterioration, vesicoureteral reflux, recurrent urinary tract infections (UTIs), and progressive damage to the transplanted kidney. If these urological issues remain unaddressed, they can result in poor graft function, increased morbidity, and, ultimately, graft loss. Thus, optimizing bladder function is essential to preserve kidney graft viability.

An assessment of bladder function is a crucial step prior to kidney transplantation, especially in patients with known or suspected lower urinary tract dysfunction. This is particularly relevant in individuals with neurogenic bladder due to spinal cord injury, myelomeningocele, or other neurological disorders, as well as those with a history of bladder augmentation, reconstructive surgeries, or severe bladder outlet obstruction. Reduced bladder compliance and capacity, when left uncorrected, can create a hostile environment for the renal graft, leading to compromised outcomes. Therefore, a thorough urological evaluation, including bladder capacity, compliance, detrusor overactivity, and voiding dynamics, should be performed before transplantation.

Video-urodynamic studies have become a standard component of pre-transplant assessment protocols in many transplant centers. These investigations allow for comprehensive visualization and quantification of bladder function, helping to stratify patients based on their risk of post-transplant urological complications. Findings such as poor compliance, high detrusor leak point pressures, or significant residual urine volumes can indicate the need for surgical intervention prior to, or simultaneously with, kidney transplantation.

Current urological interventions to address bladder dysfunction before renal transplantation include bladder augmentation (using bowel segments), urinary diversion (such as ileal conduits), and, in some cases, continent urinary reservoirs. However, for patients with irreparable bladder damage or those unsuitable for reconstructive procedures, the concept of bladder transplantation, either alone or in combination with kidney transplantation, is gaining attention. However, although this is still experimental and largely limited to preclinical studies, bladder transplantation offers a promising solution for restoring lower urinary tract function in conjunction with kidney grafting.

Recent research has explored the feasibility of bladder transplantation in animal models, addressing challenges such as vascularization, immunological acceptance, and functional integration of the transplanted bladder. Tissue engineering and regenerative medicine approaches, including the use of bioengineered bladder scaffolds seeded with autologous cells, are also being investigated as alternatives to conventional augmentation techniques. While these strategies remain investigational, they hold potential for clinical translation in the future, particularly for patients with severe bladder dysfunction where conventional options are inadequate.

Despite limited clinical experience with simultaneous bladder–kidney transplantation in humans, the theoretical benefits are compelling. By providing a compliant, low-pressure reservoir for urine storage and ensuring efficient voiding or diversion, SBKT could significantly improve kidney graft survival and overall patient outcomes in a highly select group of patients. However, further research is required to establish standardized protocols, refine surgical techniques, and address the immunological challenges associated with dual-organ transplantation.

To summarize, the interplay between bladder function and kidney graft survival underscores the importance of comprehensive lower urinary tract evaluation prior to renal transplantation. For patients with severe, untreatable bladder dysfunction, simultaneous bladder–kidney transplantation may represent a necessary and viable solution to ensure long-term graft success. The integration of rigorous pre-transplant assessment protocols, such as video-urodynamics, alongside emerging reconstructive and transplant strategies, will be essential in advancing this field and improving outcomes for complex urological-renal transplant patients.

## 6. Ethical and Social Implications

The ethics of urological transplantation encompass a wide range of complex and interrelated issues, including donor availability, consent, long-term health outcomes, social acceptance, and the equitable distribution of healthcare resources [72,74]. As advancements in this field continue to push the boundaries of medical science, it becomes increasingly important to address these ethical and social challenges proactively.

Kidney transplantation, as a life-saving procedure for patients with end-stage renal disease, is generally well-accepted both medically and socially (Table 3). However, despite its established role in healthcare, significant ethical questions persist, particularly concerning organ allocation and donor availability. The demand for kidney transplants far exceeds the available supply, leading to difficult decisions regarding prioritization [48,75]. Policies governing the distribution of kidneys often strive to balance factors such as medical urgency, compatibility, and waiting time, but disparities remain, especially when socioeconomic status and geographic location influence access to transplantation [49,125].

Living donation, which has played a crucial role in addressing kidney shortages, introduces additional ethical challenges (Table 3). While it offers a practical solution to the scarcity of organs, it also raises concerns about coercion and exploitation [126,127]. Family members, for instance, may feel obligated to donate even when they are not entirely willing. In regions where financial incentives are permitted, the risk of exploiting vulnerable populations becomes a pressing ethical issue [128,129]. Additionally, the practice of cross-border organ trafficking not only violates human rights but also undermines the integrity of legitimate transplant systems.

Bladder transplantation, in contrast to kidney transplantation, remains largely experimental and faces far more profound ethical dilemmas (Table 3). Since bladder transplantation primarily aims to improve the quality of life rather than save it, some argue that it should not take precedence over procedures with life-saving potential [130]. This perspective complicates decision-making, particularly in healthcare systems with limited resources, where cost-effectiveness and clinical benefits must be carefully weighed. Moreover, the experimental status of bladder transplantation means that long-term outcomes are still uncertain, making the informed consent process inherently more complicated. Patients must fully comprehend the risks, potential benefits, and lack of robust evidence when agreeing to undergo such a procedure. Ensuring that consent is genuinely informed requires transparent communication from medical professionals, emphasizing the experimental nature and possible complications.

Social acceptance of urological transplantation is another critical aspect that varies widely across cultures and communities. While kidney transplantation is broadly embraced due to its established success and life-saving potential, bladder transplantation might face greater societal scrutiny. The public may question whether the benefits justify the risks, especially given the challenges of restoring full bladder function and maintaining graft viability over time.

Furthermore, cultural attitudes toward organ donation, particularly those involving organs linked to personal identity or bodily functions, can significantly influence perceptions of these procedures.

Religious beliefs also play an influential role in shaping societal attitudes toward transplantation. In some communities, there may be resistance to transplanting organs associated with urinary functions, as these may be perceived as integral to personal dignity or spiritual purity. Addressing these perspectives requires an open dialogue with the community and religious leaders to foster understanding and build acceptance.

A fundamental ethical challenge lies in ensuring equitable access to advanced transplantation techniques. As medical innovations progress, disparities in healthcare access become more pronounced. Wealthier patients or those living in countries with highly developed healthcare systems are more likely to benefit from cutting-edge techniques, while disadvantaged populations may be left behind. To mitigate this gap, it is crucial to develop robust policy frameworks that ensure a fair distribution of healthcare resources and a consistent application of ethical principles, regardless of socioeconomic or geographic differences.

**Table 3:** Ethical and social implications of the ethics of urological transplantation.

Domain	Ethical/Social Dimensions	Critical Reflections	Solutions	Ref.
Transplant Ethics	Scarcity, consent, equity, legitimacy	Ethics must co-evolve with biotechnology to reconcile innovation with distributive justice	Develop adaptive, ethically responsive policy frameworks and continuous ethical oversight	[131,132]
Kidney Transplantation	Allocation ethics, structural disparities	Normative acceptance masks systemic inequities.	Refine allocation algorithms to incorporate equity metrics; enhance donor recruitment campaigns	[46,133]
Living Donation	Autonomy vs. coercion, commodification	Voluntariness is often compromised by relational or economic pressures	Enforce rigorous consent protocols, prohibit financial inducements, and strengthen donor protection laws	[125,134]
Bladder Transplantation	Non-vitality, experimental status, consent burden	Risk–benefit calculus is ethically complex in non-life-saving interventions	Prioritize transparency; establish a specialized ethics review for quality-of-life transplant trials	[135,136]
Cultural Reception	Identity, embodiment, and acceptability	Deeply embedded symbolic meanings affect social legitimacy	Initiate culturally sensitive public engagement; include anthropologists and sociologists in policy design	[48,137]
Religious Paradigms	Purity, dignity, moral authority	Religious resistance may impede the implementation	Foster interfaith dialogues; incorporate theological perspectives into bioethical consultation	[134,138]
Global Health Justice	Access asymmetry, systemic bias	Biomedical innovation risks entrenching global disparities	Create equitable access policies; support international transplant cooperation and capacity-building	[139,140]
Emerging Frontiers	Xenotransplantation, gene editing, and synthetic biology	These modalities destabilize classical bioethical categories	Establish anticipatory ethics frameworks; integrate risk ethics and public deliberation mechanisms	[131,141]
Strategic Imperative	Normative adaptability, stakeholder inclusion	Ethical governance must remain reflexive and pluralistic	Institutionalize interdisciplinary ethics committees; maintain open, iterative dialogue across sectors	[142,143]

As xenotransplantation advances from experimental models to early clinical trials, understanding public and patient attitudes has become essential to guiding ethical implementation, regulatory oversight, and equitable access. Recent studies reveal a complex and nuanced public perspective that is shaped by knowledge gaps, ethical concerns, cultural influences, and trust in the medical system.

In the largest national survey to date, Padilla et al. (2024) assessed the attitudes of over 5000 U.S. adults toward xenotransplantation and found that although 36% were open to receiving a pig organ if needed, nearly 40% expressed discomfort, and more than half cited lack of evidence or fear of complications as their primary concern [18]. Notably, younger individuals, women, racial minority groups, and those without a current transplant need were more likely to express hesitancy. These findings underscore a critical knowledge and trust gap that must be addressed before broader public acceptance can be achieved.

Additional insights come from studies focused on specific populations. Padilla et al. (2020) explored attitudes among parents of children with congenital heart disease and their healthcare providers [144]. While overall acceptance was relatively high, especially among physicians (86%), religious influence and discomfort with using pig hearts as bridge organs negatively impacted acceptance, particularly among parents. This highlights the need for stakeholder-specific education and sensitivity to personal values in decision-making around xenotransplantation.

Hurst et al. (2024) address a critical gap in the emerging field of xenotransplantation by outlining key ethical considerations for pediatric trials [145]. Given the severe shortage of pediatric donor hearts and high waitlist mortality, neonates and infants with complex congenital heart disease may benefit significantly from xenografts, either as definitive therapy or as a bridge to allotransplantation. A central issue raised is whether XTx trials should begin in adults or children. While adult-first trials are standard, the authors argue that, in select pediatric cases, the risk–benefit ratio may favor early pediatric inclusion, particularly for patients lacking other viable options.

A systematic review by DeLaura et al. (2023) further consolidated attitudes among patients with end-stage renal disease or kidney transplants [146]. While 63% were willing to accept a xenograft with an equivalent function to an allograft, willingness dropped sharply for grafts with inferior function or those intended as temporary solutions. The concerns most frequently cited included infection risk, graft performance, social stigma, and animal welfare, highlighting the multifaceted nature of public perception.

Overall, these studies suggest that although there is a baseline level of support for xenotransplantation, especially in life-threatening situations, public attitudes remain cautious. Success in this field will depend not only on scientific and clinical milestones but also on transparent public dialogue, culturally sensitive education, and trust-building measures. Addressing ethical concerns, particularly around animal use and informed consent, and ensuring diverse representation in clinical trials will be critical to the responsible rollout of xenotransplantation in human medicine.

Looking to the future, as research into urological transplantation advances, new ethical questions are likely to emerge. Innovations such as xenotransplantation and bioengineered organs offer promising solutions to donor shortages but also raise profound ethical concerns related to animal rights, genetic modification, and human safety. Additionally, novel approaches to immunosuppression and gene editing might challenge the established ethical norms, calling for updated guidelines and policies. To address these evolving challenges, it is essential to maintain a dynamic and inclusive ethical discourse. Stakeholders, including ethicists, healthcare professionals, researchers, patients, and policymakers, must collaborate to establish transparent standards that balance scientific progress with moral and social responsibility. As urological transplantation continues to evolve, maintaining this dialogue will be critical to ensuring that innovation is pursued responsibly and ethically.

## 7. Challenges and Future Prospects

The future of urological transplantation holds immense promise, driven by continuous advancements in medical science, biotechnology, and regenerative medicine. The overarching goals remain centered on enhancing graft survival, minimizing immune rejection, and improving functional outcomes, particularly for complex transplants such as the bladder [147,148]. As research progresses, several emerging technologies and scientific breakthroughs have the potential to revolutionize the field, addressing the persistent challenges that have hindered widespread clinical application.

One of the most transformative areas of development is gene editing, particularly through technologies such as CRISPR-Cas9 and other genome-modifying platforms. These tools offer unprecedented precision in modifying donor organs to reduce immunogenicity and enhance compatibility, potentially mitigating the risk of acute and chronic rejection [149]. Genetic engineering could also be applied to modify recipient immune responses, fostering long-term graft tolerance without the need for lifelong immunosuppressive therapy [150,151]. Furthermore, the application of gene-edited animal organs, particularly from genetically modified pigs, is being actively explored as a solution to the chronic shortage of human donors.

However, before widespread clinical adoption, it will be critical to address the ethical concerns, ensure safety in human trials, and overcome the remaining immunological barriers associated with xenotransplantation [152,153]. Gene editing in human transplantation raises questions about long-term consequences, potential off-target effects, and the moral implications of genome manipulation. Xenotransplantation introduces additional controversies, including zoonotic disease transmission risks, animal welfare considerations, and unresolved immunological challenges. Rigorous regulatory frameworks and extensive clinical validation are essential before these technologies can achieve widespread acceptance.

Tissue engineering and regenerative medicine represent another frontier with the potential to redefine urological transplantation [154]. Advances in bioprinting and stem cell-based therapies are paving the way for bio-artificial organs that closely replicate the structure and function of native tissues [155]. The ability to engineer kidneys and bladders tailored to individual patients using their own stem cells could drastically reduce waiting times, eliminate the risk of immune rejection, and improve long-term outcomes [47,73]. One of the most significant challenges in bladder transplantation is ensuring adequate vascularization and innervation to maintain proper urinary function. Researchers are focusing on developing vascularized and innervated bladder constructs, incorporating biomimetic scaffolds and growth factor delivery systems to promote functional integration after transplantation [156,157]. Techniques such as 3D bioprinting and stem cell-derived bio-artificial organs offer the prospect of patient-specific grafts, potentially eliminating immune rejection and reducing waitlist dependence. Despite their promise, these technologies are still in their developmental stages, facing hurdles in scalability, reproducibility, and long-term functional outcomes. The complexity of replicating the intricate architecture and dynamic functions of native urological tissues limits immediate clinical translation. Furthermore, ethical concerns related to stem cell sourcing and manipulation persist, necessitating transparent guidelines and societal dialogue.

Beyond the technological innovations, immunomodulation remains a cornerstone of improving transplant outcomes. Traditional immunosuppressive therapies, while effective, come with considerable side effects, including nephrotoxicity, increased infection risk, and metabolic complications. Novel strategies such as tolerogenic cell therapies, which induce immune tolerance without requiring continuous pharmacological suppression, are showing significant promise [59,158]. The use of regulatory T cells (Tregs), mesenchymal stem cells, and other immune-regulatory approaches may allow for long-term graft survival while reducing the burden of systemic immunosuppression [159]. Additionally, advances in biomarker discovery, particularly urine-based assays and donor-derived cell-free DNA (dd-cfDNA) monitoring, are facilitating the early detection of rejection episodes, enabling more personalized and pre-emptive intervention strategies [160,161]. Indeed, the integration of biomarker-based monitoring, including donor-derived cell-free DNA and urine assays, offers a pathway for personalized immunosuppression management. However, the clinical utility and cost-effectiveness of these tools in routine practice remain subjects of ongoing investigation and debate.

Artificial intelligence (AI) and machine learning are also poised to play a crucial role in optimizing transplant medicine. Predictive analytics can enhance donor–recipient matching by analyzing vast amounts of histocompatibility and immunological data, allowing for a better risk assessment and improved graft survival [162,163]. AI-driven monitoring systems are being developed to track post-transplant biomarkers, detect early signs of rejection, and recommend personalized immunosuppressive adjustments, ultimately improving patient outcomes and reducing complications [164,165]. Nonetheless, the adoption of AI introduces ethical and practical concerns, including data privacy, algorithmic transparency, and the risk of over-reliance on automated systems. Ensuring that AI complements rather than replaces clinical judgment is critical to maintaining trust and ensuring patient-centered care.

Despite technological progress, major challenges remain in developing reliable, functional bladder grafts. The bladder’s unique anatomy and physiology, requiring precise neural integration, a strong blood supply, and dynamic compliance, create significant obstacles. Restoring continence, compliance, and coordinated voiding is still an unmet goal. Overcoming these barriers demands close collaboration among urology, transplant surgery, immunology, genetics, bioengineering, and bioethics experts. Rigorous validation through preclinical studies and clinical trials is essential to ensure safety and effectiveness before clinical application.

While the future of urological transplantation is marked by exciting technological advancements, the field continues to face several critical barriers that must be addressed to enable clinical translation. For example, although xenotransplantation, particularly using genetically modified pig organs, holds promise for alleviating organ shortages, it remains entangled in regulatory, ethical, and public concerns [166,167]. Notably, questions about zoonotic disease transmission (such as porcine endogenous retroviruses), animal welfare, and the long-term immunological compatibility of xenografts have provoked ongoing debate. Regulatory agencies, including the FDA and EMA, continue to exercise caution, and broader societal acceptance remains uncertain. These concerns highlight the need for not only scientific breakthroughs but also transparent regulatory frameworks and public engagement strategies.

Similarly, while bioengineered organs such as 3D-printed bladders and stem cell-derived constructs offer tremendous theoretical advantages, including patient-specific grafts and reduced rejection, scalability and reproducibility remain significant limitations. Manufacturing fully functional, vascularized, and innervated urological grafts that can withstand physiological pressures is still a complex challenge. Furthermore, the high cost and technical expertise required for large-scale production raise questions about economic feasibility and global accessibility, particularly in low-resource settings. Without standardized manufacturing protocols and regulatory harmonization, widespread adoption will remain limited.

The integration of AI in transplantation introduces its own set of challenges [168]. While AI systems can significantly enhance donor–recipient matching and post-transplant monitoring, implementation costs and infrastructure demands may limit adoption in many centers [169]. In addition, issues of algorithmic bias, data privacy, and lack of explainability can hinder clinical acceptance. For example, trust in AI-driven recommendations can be undermined if clinicians cannot understand or verify the rationale behind algorithmic decisions [128]. Balancing automation with human clinical judgment and ensuring equitable access to AI tools are crucial considerations moving forward.

Addressing these challenges requires not only technological innovation but also cross-disciplinary collaboration, robust regulatory oversight, and sustained investment in translational research. Public health systems, policymakers, and the medical community must work together to ensure that cutting-edge innovations in transplantation do not remain confined to the laboratory or elite institutions but are translated into safe, ethical, and widely available therapies.

## 8. Conclusions

This review underscores the remarkable progress achieved in the field of urogenital transplantation, particularly in kidney transplantation, while highlighting the persistent challenges and future prospects for both kidney and bladder grafts. Kidney transplantation has become the gold standard for treating end-stage renal disease, offering substantial improvements in patient survival and quality of life. Advances in surgical methods, immunosuppressive regimens, and organ preservation technologies have contributed to better short- and long-term outcomes. Nonetheless, significant challenges remain, including the ongoing shortage of donor organs, risks of chronic graft dysfunction, and the adverse effects associated with lifelong immunosuppression. Emerging strategies such as precision immunotherapy, machine perfusion, and xenotransplantation—especially with genetically engineered porcine kidneys—show promise for expanding the donor pool and further improving outcomes.

In contrast, bladder transplantation is still in the experimental stage and faces considerable obstacles. Successful transplantation requires not only vascularization but also the functional integration of nerves and smooth muscle, which are essential for restoring normal bladder function. While preclinical studies and advances in tissue engineering, regenerative medicine, and robotic surgery have demonstrated technical feasibility, clinical translation is limited by unresolved issues in tissue integration, immune response, and long-term graft viability. Additionally, ethical, regulatory, and logistical challenges, particularly in the context of xenotransplantation and bioengineered organs, must be addressed before these approaches can become routine clinical practice.

Looking ahead, interdisciplinary collaboration will be vital to overcoming these barriers. Continued research in immunomodulation, bioengineering, and organ preservation, as well as the development of personalized immunosuppressive protocols and improved graft monitoring, will be essential for optimizing both kidney and future bladder transplantation outcomes. As these innovations progress from the laboratory to the clinic, they hold the potential to transform the management of end-stage urogenital organ failure and significantly enhance patient care.

## Data Availability

No new data were created or analyzed in this study.
